# Regulation of myogenic activation of p38 MAPK by TACE-mediated TNFα release

**DOI:** 10.3389/fcell.2014.00021

**Published:** 2014-05-23

**Authors:** Yi-Ping Li, Airu Niu, Yefei Wen

**Affiliations:** Department of Integrative Biology and Pharmacology, University of Texas Health Science CenterHouston, TX, USA

**Keywords:** muscle regeneration, satellite cells, differentiation, p38 MAPK, TNFα, TACE

## Abstract

The activation of p38 MAPK in myogenic precursor cells (MPCs) is a key signal for their exit of cell cycle and entry of the myogenic differentiation program. Therefore, identification of the signaling mechanism that activates p38 MAPK during this process is important for the understanding of the regulatory mechanism of muscle regeneration. This article reviews recent findings regarding the role of inflammatory cytokine tumor necrosis factor-α (TNFα) as a key activator of p38 MAPK during myogenesis in an autocrine/paracrine fashion, and the signaling mechanisms that converge upon TNFα converting enzyme (TACE) to release TNFα from differentiating MPCs in response to diverse regenerative stimuli.

Skeletal muscle regeneration is vital for the maintenance of muscle homeostasis, the repair of injury and the adaptation to training. Skeletal muscle regeneration requires the re-initiation of the myogenic program that involves the sequential expression or repression of such transcription factors as Six1/4, Pax3/7, Myf5, MyoD, myogenin, and MRF4 to turn on muscle-specific gene expression in myogenic precursor cells (MPCs), known as satellite cells (Bentzinger et al., 2012). The execution of the myogenic program requires epigenetic regulations that control the activity of the aforementioned transcription factors precisely to establish and maintain the myogenic identity in quiescent MPCs and to enable the proper response to environmental cues in activated MPCs (Giordani and Puri, 2013).

A key player in epigenetic regulation of the myogenic program is p38 MAPK. Activation of p38 MAPK is essential for the initiation of myogenic differentiation in myoblasts and embryo (Cuenda and Cohen, 1999; Zetser et al., 1999; Puri et al., 2000; Wu et al., 2000; Cabane et al., 2003; Penn et al., 2004; de Angelis et al., 2005). Activated p38 MAPK mediates the transition of proliferating satellite cells into terminal differentiation and ensuing muscle-specific gene expression through multiple actions including the initiation of cell cycle exit by down-regulating Pax7, the activation of key myogenic transcription factors MyoD and MEF2, and the activation of chromatin remodeling to allow access of MyoD and MEF2 to the myogenic loci (Lluis et al., 2006; Giordani and Puri, 2013). In addition, the myogenesis-promoting role of p38 MAPK has been linked to its α subtype specifically (Cabane et al., 2003; Perdiguero et al., 2007; Palacios et al., 2010). Thus, p38 MAPK is considered a molecular switch for the activation of myogenic differentiation, and the signaling mechanism that mediates myogenic activation of p38 MAPK is of fundamental importance for myogenic gene expression. However, for a long period of time the signaling mechanism that mediates p38 MAPK activation in satellite cells during muscle regeneration was poorly understood. This review summarizes the recent literature that depicts a key role of TACE-mediated release of TNFα from MPCs in the activation of p38 MAPK during muscle regeneration.

## Inflammation is critical to muscle regeneration

Myogenic activation of p38 MAPK is controlled either by the developmental program, as in embryonic myogenesis, or by environmental input, as in adult myogenesis associated with muscle regeneration. Muscle regeneration takes place in a highly inflammatory environment. Inflammation has been recognized as a key response to muscle injury required for muscle regeneration (Tidball, 1995, 2005). Infiltrated inflammatory cells, particularly macrophages, facilitate muscle regeneration via phagocytosis of cellular debris and release of soluble factors that promote satellite cell proliferation and differentiation (Cantini et al., 1994; Cantini and Carraro, 1995; Chazaud et al., 2003). Among those soluble factors, there are not only chemoattractants and growth factors that are traditionally recognized as factors promoting muscle regeneration but also cytokines that are known mainly as inflammatory mediators including TGFβ, leukemia inhibitory factor (LIF) and IL-6 (Hawke and Garry, 2001; Charge and Rudnicki, 2004).

All the growth factors involved in muscle regeneration including fibroblast growth factor (FGF), insulin-like growth factor (IGF-I), hepatocyte growth factor (HGF), epidermal growth factor (EGF), and platelet-derived growth factor (PDGF) promote the proliferation of satellite cells. Most of them also inhibit the differentiation of satellite cells. Only IGF-I has been convincingly shown to promote both the proliferation and differentiation of satellite cells (Hawke and Garry, 2001; Charge and Rudnicki, 2004). However, IGF-I is not able to activate p38 MAPK or to induce myogenesis when p38 MAPK is inhibited (Wu et al., 2000). Some of the cytokines including TGFα, LIF (Austin et al., 1992), and IL-6 (Cantini et al., 1995; Munoz-Canoves et al., 2013) also promote satellite cell proliferation. On the other hand, TGFβ inhibits differentiation (Massague et al., 1986; Olson et al., 1986). However, the inflammatory cytokine TNFα has emerged as a key mediator of myogenic differentiation due to its activation of p38 MAPK.

## TNFα receptor-mediated signaling is required for myogenesis

Several lines of evidence support a central role of TNFα in the activation of adult myogenesis. First, muscle regeneration takes place in an environment with elevated TNFα levels. Mammalian muscle constitutively synthesizes TNFα (Saghizadeh et al., 1996). Coinciding with the onset of muscle regeneration, TNFα level in injured muscle rises dramatically because of a strong increase in TNFα synthesis by injured myofibers as well as TNFα release by infiltrating inflammatory cells (Tews and Goebel, 1996; De Bleecker et al., 1999; Zador et al., 2001; Warren et al., 2002). Importantly, myofiber synthesis of TNFα is positively correlated to regenerating activity (Kuru et al., 2003). At the same time, there is an increase in TNFα receptor expression in injured muscle fibers (De Bleecker et al., 1999; Zador et al., 2001), suggesting a physiological role for TNFα receptor-mediated signaling in injured muscle. Further, TNFα is constitutively expressed in C2C12 myoblasts and a rapid increase of TNFα expression takes place during the early hours of differentiation, which is critical to muscle-specific gene expression, suggesting that TNFα regulates myogenesis in an autocrine/paracrine fashion (Li and Schwartz, 2001). These data indicate an orchestrated effort by myocytes and inflammatory cells to increase TNFα receptor-mediated signaling in MPCs during muscle regeneration.

Second, TNFα receptor-mediated signaling is critical to myogenesis in diverse muscle regeneration models. TNFα receptor double-knockout (p55^−^/^−^p75^−/−^) impairs strength recovery of mouse muscle injured by freezing, which suggests the participation of TNFα in the regulation of muscle regeneration (Warren et al., 2002). In addition, it was observed in cardiotoxin-injured mouse muscle that TNFα receptor double-knockout increases the expression of Cyclin D1 and decreases the expression of p21 as well as the activation of MEF2C, resulting in a blockade of myogenin expression and impairment of muscle regeneration (Chen et al., 2005). These data suggest that TNFα receptor-mediated signaling is required for proliferating satellite cells to exit cell cycle and enter terminal differentiation. Subsequent *in vitro* studies confirmed this role of TNFα. In cultured myoblasts with TNFα receptor double-knockout the activation or expression of early myogenic markers MEF2C, p21 and myogenin are blocked, resulting in decreased expression of myosin heavy chain (MHC), when differentiation is induced by either serum withdraw (Chen et al., 2007) or mechanical stretch (Zhan et al., 2007). Further, addition of a TNFα-neutralizing antibody to the culture medium of myoblasts recapitulates a critical role of myoblast-released autocrine TNFα in the activation of myogenic differentiation. Conversely, addition of exogenous TNFα to myoblast cultures, which mimics the elevated levels of TNFα found in injured muscle, further increases myoblast differentiation (Chen et al., 2007). These observations support the concept that combined TNFα release from myocytes and inflammatory cells in injured muscle promotes myogenesis. A more recent study by Palacios and colleagues further demonstrated that neutralizing TNFα in mdx mice blocks myogenesis by interfering with differentiation-associated repression of Pax7 levels which is essential for cell cycle exit and the progression of activated satellite cells in myogenic lineage (Palacios et al., 2010). Therefore, TNFα receptor-mediated signaling has a central role in the regulation of the exit of cell cycle and the initiation of myogenic differentiation in satellite cells.

Third, TNFα receptor-mediated signaling promotes myogenic differentiation through its activation of p38 MAPK. TNFα is one of the many activators of p38 MAPK (Zarubin and Han, 2005). In myocytes TNFα receptor-associated factor 6 (TRAF6) mediates p38 MAPK activation through Transforming growth factor β activated kinase-1 (TAK1) (Xiao et al., 2012). However, whether TNFα receptor-mediated signaling is critical to myogenic activation of p38 MAPK was unknown until such a role has been demonstrated in diverse models of muscle regeneration. In cardiotoxin-injured mouse muscle TNFα receptor double-knockout blocks the activation of p38 MAPK (Chen et al., 2005). Neutralizing TNF in mdx mice blocks p38 MAPK activation in regenerating mdx muscle (Palacios et al., 2010). Consistent to the *in vivo* findings, in cultured myoblasts TNFα receptor double-knockout or treatment with a TNFα-neutralizing antibody blocks p38 MAPK activation, resulting in a blockade of myogenic differentiation similar to the effect of the pharmacological inhibitor of p38 MAPK, SB203580 (Chen et al., 2007; Zhan et al., 2007). Further, the activation of p38 MAPK has been shown to be essential to TNFα receptor-mediated signaling to promote myogenic differentiation. Forced activation of p38 MAPK by the expression of a constitutively active MKK6 (MKK6bE) in the muscle of TNFα receptor double-knockout mice rescues impaired myogenesis and muscle regeneration (Chen et al., 2007). These observations indicate that TNFα receptor-mediated signaling promotes myogenesis through the activation of p38 MAPK.

## TACE-mediated autocrine TNFα release from myoblasts activates p38 MAPK and myogenesis

TNFα is synthesized as a 26 kDa transmembrane pro-protein and released as a 17 kDa free peptide into extracellular space upon cleavage primarily by TNFα converting enzyme (TACE). TACE, also known as A disintegrin and metalloprotease (ADAM) 17, is a ubiquitous transmembrane protein that belongs to the ADAM family of disintegrin metalloproteinases (Blobel, 1997; Black, 2002). The cleavage of TNFα from the plasma membrane by TACE allows the release of free TNFα in muscle from infiltrating inflammatory cells, primarily macrophages, as well as myocytes as a paracrine or autocrine regulator. TACE activity is regulated posttranscriptionally by cellular signaling events (Zhang et al., 2000, 2001; Fan et al., 2003), hence, TACE could be a rate-limiting regulator of TNFα-mediate signaling in myogenesis. Importantly, in cardiotoxin-injured muscle, there is not only an increase in TNFα synthesis but also an increase in TNFα cleavage, which starts within 1 day and reaches the peak level around day 3 of injury, coinciding with the activation of satellite cells and the initiation of myogenic differentiation (Chen et al., 2007). TNFα was also shown released by cultured proliferating myoblasts at a low level, which is significantly increased upon differentiation and lasts for at least 2 days, when myogenic gene expression is being initiated (Chen et al., 2007). Neutralizing the TNFα released into the culture medium blocks p38 MAPK activation during myoblast differentiation induced by either serum-withdraw (Chen et al., 2007) or mechanical stretch (Zhan et al., 2007). Further, a dramatic increase in TACE activity is observed in differentiating myoblasts, which is rate limiting for the activation of p38 MAPK and subsequent myogenic differentiation (Zhan et al., 2007). Therefore, TACE-mediated autocrine TNFα release from myoblasts activates p38 MAPK and myogenesis.

## TACE activity in myoblasts is regulated by distinct signaling mechanisms specific to the nature of myogenic stimulus

Given that TACE-mediated autocrine TNFα release is a key step for the activation of p38 MAPK in differentiating myoblasts, there must be intrinsic mechanisms that control the release of TNFα through regulating TACE activity in response to myogenic stimuli. TACE activity is normally repressed by its physiological inhibitor, tissue inhibitor of metalloproteinase 3 (TIMP3), a member of the tissue inhibitor of metalloproteinase family that plays an important role in the regulation of inflammation (Black, 2004). TIMP3 is the only one of four TIMPs that binds to the extracellular matrix (Mohammed et al., 2003) and inhibits TACE activity through chelating the extracellular active-site zinc with its N-terminus (Gomis-Rüth et al., 1997; Amour et al., 1998; Lee et al., 2005). Notably, TIMP3 is constitutively expressed in muscle cells (Leco et al., 1994), which suggests a physiological role for TIMP3 in muscle cells. In searching for the mechanisms that regulate TACE activity during myogenesis, Liu et al. (2010) recently discovered that in mouse muscle undergoing regeneration induced by cardiotoxin injury or functional overloading, TIMP3 expression at mRNA and protein levels is dramatically down-regulated within 1 day, and then recovered to normal level when regeneration is complete. The down-regulation of TIMP3 takes place in activated satellite cells. Similar down-regulation of TIMP3 is observed in cultured myoblasts that are differentiating in low-serum medium. Ectopic expression of TIMP3 in myoblasts or muscle that counters TIMP3 down-regulation blocks p38 MAPK activation and myogenic differentiation. Further, TIMP3 down-regulation in differentiating myoblasts is mediated by microRNA-206, a myogenesis-promoting microRNA that is induced during myoblast differentiation (Kim et al., 2006; Rao et al., 2006). Thus, constitutively expressed TIMP3 acts as a gatekeeper of myogenic differentiation that helps to prevent premature differentiation and maintain the satellite cell pool. The down-regulation of TIMP3 opens the gate of differentiation by allowing TACE to release TNFα that activates p38 MAPK.

However, the down-regulation of TIMP3, which requires many hours to take place, does not explain the rapid activation of p38 MAPK by TNFα observed in mechanically stretched myoblasts (Zhan et al., 2007). More recently, Niu and colleagues described a signaling mechanism of rapid activation of TACE in muscle in response to mechanical loading involving the non-receptor tyrosine kinase Src. Src plays an important role in the transduction of mechanical stimulation into biochemical signaling in a variety of load-sensitive cells (Han et al., 2004; Wang et al., 2005). In cells that are mechanically stimulated, Src undergoes phosphorylation and dephosphorylation rapidly, which activates Src. Activated Src in turn activates its downstream effectors through tyrosine phosphorylation (Sai et al., 1999). Niu et al. (2013) discovered that Src is rapidly activated in mechanically stretched myoblasts. Src then quickly phosphorylates TACE that possesses the structural features of Src substrates in its intracellular tail including a potential SH3-binding motif (P^731^APQTPGR^738^) adjacent to a putative tyrosine phosphorylation motif (K^696^KLDKQYESL^705^). Utilizing an antibody raised against this motif with phosphorylated Tyr-702 residue, these authors demonstrated that Src phosphorylates Tyr-702 upon mechanical stimulation, which activates TACE. Expression of a TACE mutant in which the Tyr-702 residue is replaced by alanine blocks TACE-activated TNFα release, p38 MAPK activation and differentiation in mechanically stretched myoblasts. Further, Src deficiency suppressed activation of p38 MAPK and myogenesis in mechanically stretched myoblasts as well as in satellite cells of overloaded soleus in mice. Consequently, overloading-induced muscle regeneration is impaired in Src-deficient mice. The presence of such stimulus-specific signaling mechanisms that regulate TACE release of autocrine TNFα from MPCs provides further support for TNFα as a key regulator of the myogenic program responsible for the activation of p38 MAPK during myogenesis.

## Other considerations

It is noteworthy that the effect of TNFα on myogenic differentiation is highly complex. The effect of TNFα on myoblast differentiation is bimodal. At physiological concentrations found in injured muscle (up to 0.05 ng/ml), exogenously added TNFα promotes myoblast differentiation. However, at higher concentrations that are seen in inflammatory diseases exogenously added TNFα inhibits myoblast differentiation, despite that its activation of p38 MAPK is intact (Chen et al., 2007). Indeed, a number a studies that employed high concentrations of TNFα reported similar inhibitory effect of TNFα on myoblast differentiation (Guttridge et al., 2000; Langen et al., 2001; Trendelenburg et al., 2012) or muscle regeneration (Moresi et al., 2008). The paradoxical effects of TNFα on myogenesis illustrate the importance of distinguishing its physiological role from its pathological roles.

Another issue of significance is that TNFα receptor-mediated signaling is not required for embryonic myogenesis. Mice with TNFα receptor double-knockout develop seemly normal muscle. Myogenic activation of p38 MAPK during development is likely mediated by the cell surface receptor Cdo (Kang et al., 2008) or by amphoterin (HMGB1) through engaging the receptor for advanced glycation end products (RAGE), whose expression is developmentally regulated (Sorci et al., 2004).

Further, p38 MAPK has been shown to promote satellite cell activation and proliferation (Jones et al., 2005) through an asymmetric activation pattern (Troy et al., 2012). However, whether TNFα is responsible for p38 MAPK activation during these events is unknown.

## Conclusions

Recent progress indicates that TACE-released autocrine TNFα from MPCs is a key mediator of p38 MAPK activation that initiates myogenic differentiation through epigenetic regulations. Emerging evidence also revealed that MPCs respond to diverse environmental cues of muscle regeneration by activating p38 MAPK through the activation of TACE release of autocrine TNFα through at least two distinct signaling pathways, namely, the down-regulation of TIMP3 by miR-206 or the activation of TACE by Src in a stimulus-dependent manner. Therefore, TACE-mediated TNFα release from MPCs plays a key role in the re-initiation of the myogenic program during muscle regeneration.

### Conflict of interest statement

The authors declare that the research was conducted in the absence of any commercial or financial relationships that could be construed as a potential conflict of interest.
